# The regeneration of Fe-EDTA denitration solutions by nanoscale zero-valent iron

**DOI:** 10.1039/c8ra08992b

**Published:** 2018-12-21

**Authors:** Wei Jiang, Xiaolong Wang, Qiang Xu, Jianbai Xiao, Xionghui Wei

**Affiliations:** College of Chemistry and Molecular Engineering, Peking University Beijing 100871 China xhwei@pku.edu.cn

## Abstract

Fe(ii) ethylenediaminetetraacetate (EDTA) chelate solution is generally considered to be an effective nitric oxide (NO) absorbent. However, since the ferrous active site is occupied by nitric oxide and the ferrous chelate is oxidized to ferric chelate by oxygen in air, its absorption capacity will gradually decrease with the NO absorption process. Here, we propose a method for regenerating the NO-attenuated Fe(ii)EDTA solution by adding nanoscale zero-valent iron (NZVI) under three different pH conditions. Furthermore, compared with the commercially available iron powder, NZVI was also found to be effective not only for the regeneration of expired Fe-EDTA solution but also for the reduction of Fe(iii) EDTA solution. According to the results obtained herein, different acidity levels of solution, from weakly acidic to near neutral, are all suitable for the regeneration–absorption process.

## Abstract 2

NZVI is very effective for the regeneration of the inactive Fe chelate solution in the NO absorption process.

## Graphical abstract

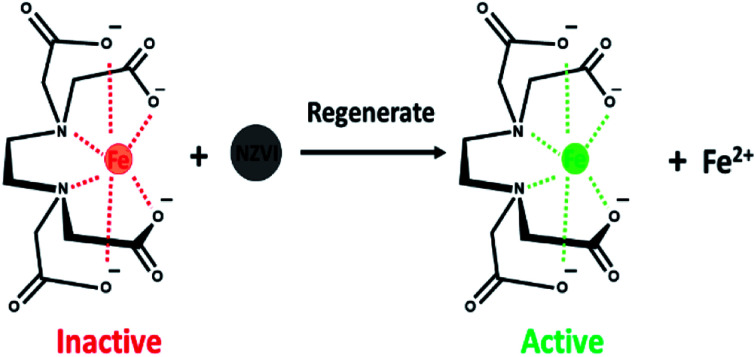

## 1. Introduction

Nitric oxide (NO) is the main component of the nitrogen oxides (NO_*x*_) present in fossil combustion power plant flue gas. The complexing absorption of NO in aqueous Fe(ii)EDTA solution is a promising method on account of its prompt absorption rate and high absorption capacity. However, it is also noticed that Fe(ii) species are continuously oxidized/occupied by O_2_/NO in the absorption process, overshadowing the solution's application from the perspective of the environment and economic cost disadvantage.^1^ Therefore, developing a method to recycle or regenerate the nitrosyl complex solution is of great interest for many research groups in this field.^1–6^

Biological reduction of NO chelated by Fe(ii)-EDTA, a novel chemical absorption–biological reduction integrated process, is regarded as a promising alternative to the conventional selective catalytic reduction process. The ferrous chelate absorbent can be regenerated by microorganisms to sustain a persistent NO removal capacity.^6–8^ However, some stringent conditions are needed to maintain the microbial growth, which is difficult to meet in many practical applications. Moreover, the regeneration rate of microorganisms serving as the absorbent is too slow to be accepted readily in an industrial scale.^7–9^ Electrochemical reduction regeneration is another promising technology under detailed investigation in recent years.^5,10,11^ It can effectively convert Fe(ii)EDTA–NO to harmless N_2_ or NH_4_^+^ and release the active ferrous chelate species. Meanwhile, the ferric chelate is reduced to ferrous chelate, and the majority of the chelate is maintained in the active ferrous state.^11^ To industrialize this technology, high-surface-area electrodes embedded with electro-generative cells must be developed to meet the large flue gas flow requirement. Additional electric energy consumption is another concern when popularizing this technology.^5,11^

Besides the two methods mentioned above, chemical reduction is also being investigated as an important traditional method to regenerate the absorption solution. Chemicals with reducibility, such as polyphenols, dithionite, hydrazine, metal powder, *etc.*, are screened and adopted directly in the absorption/regeneration process.^3,4,12–17^ Feiqiang *et al.* investigated the mechanism and kinetics of Fe(ii)EDTA–NO and Fe(iii)EDTA reduction by iron powder.^15,16^ Adam *et al.* found that zinc powder can effectively reduce Fe(iii)EDTA aqueous solution at 20 °C, with a noticeably higher reduction rate compared to aluminum and tin powder. In addition, the reduction capability of nanoscale zinc powder was about 11 times higher than that of normal-sized zinc.^3,18^ Recently, nanoscale zero-valent iron technology has been extensively adopted in wastewater treatment.^19–24^ This perspective of applying nanoscale chemicals to water treatment encouraged us to develop NZVI technology to regenerate the absorption liquid.

In this paper, we extended the application of NZVI in waste treatment and demonstrated that NZVI could effectively regenerate NO-saturated Fe(ii)EDTA solution. We also found that NZVI has a higher reactivity than commercially available iron powder for the reduction of oxidized Fe(ii)EDTA solution.

## 2. Experimental section

### 2.1. Chemicals

Ferrous sulfate heptahydrate (FeSO_4_·7H_2_O), ferric nitrate nonahydrate (Fe(NO_3_)_3_·9H_2_O), ethanol, polyethylene glycol 600 (PEG-600), and tetrasodium ethylenediaminetetraacetate (Na_4_EDTA·2H_2_O) were analytical grade and purchased from Sinopharm Chemical Reagent Co., Ltd. Sodium borohydride (NaBH_4_, 95% purity) was purchased from Guangdong Guanghua Sci-Tech Co., Ltd. Reduced iron powder (98% purity) was purchased from Xilong Scientific Co., Ltd. Sodium acetate (AR) and acetic acid (AR) were used to prepare the 0.2 M HAc-NaAc buffer solution. All reagents were used without further purification. NO (1000 ppm, v/v, with N_2_ balanced) and N_2_ (99.9% purity) were purchased from Beijing Nanfei Gongmao, Ltd.

### 2.2. Preparation of iron chelate solution

The 0.02 M Fe(ii)EDTA solution was freshly prepared with equal mol FeSO_4_ and Na_4_EDTA at desired acidity, adjusted by diluted NaOH and HCl and recorded by pH meter. The 0.02 M Fe(iii)EDTA solution was prepared with Fe(NO_3_)_3_ and Na_4_EDTA of the same molar ratio at the desired pH. The expired Fe-EDTA solution was prepared by exposing the freshly made Fe(ii)-EDTA solution (0.02 M) to air in a transparent jar for three days. All the solutions were prepared with deionized water.

### 2.3. Preparation of nanoscale zero-valent iron

3.66 g FeSO_4_·7H_2_O, 2 g PEG-600, 20 ml ethanol, and 100 ml water were used to prepare the Fe^2+^-containing solution. PEG-600 was used as stabilizer to produce stabilized NZVI and weaken the tendency of agglomeration, which is a thermodynamically favorable process for the metal nanoparticles.^24,25^ Then, 0.91 g NaBH_4_, 0.1 g NaOH and 100 ml water were used to prepare the reducing solution. In the next step, NZVI was synthesized by reducing Fe^2+^ with NaBH_4_ solution, added dropwise at room temperature with magnetic stirring. The reaction can be described by the following equation:

1 Fe^2+^ + 2BH^−^_4_ + 6H_2_O → Fe + 2B(OH)_3_ + 7H_2_↑

After complete reaction, the wet NZVI was separated and collected from the black suspension using magnetic separation, and it was washed with water and ethanol three times each. Prior to being characterized and fed to the absorbent, the metal particles were dried in a vacuum container at room temperature for 24 h, then ground into powder with the agate mortar.

### 2.4. Apparatus and procedure of nitric oxide absorption

**Fig. 1:**
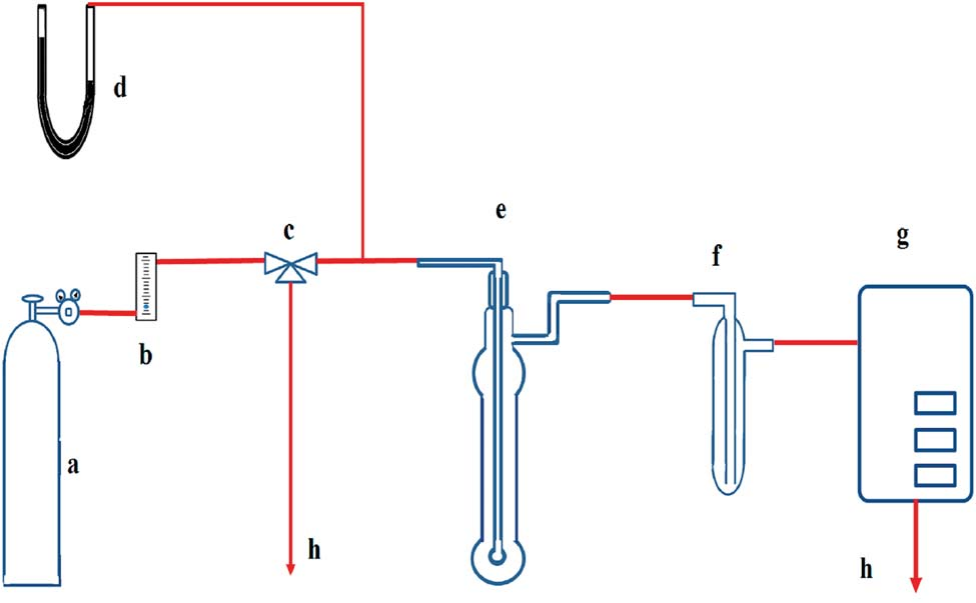
Schematic diagram of the experimental setup for the removal of NO. (a) NO/N_2_ cylinder, (b) rotor flow meter, (c) three-way valve, (d) mercury pressure indicator, (e) glass bubbling reactor, (f) ice-water bath, (g) gas measure analyzer, (h) tail gas treatment.

The schematic diagram of the absorption experiment is shown in Fig. 1. The basic bubbling apparatus consists of a lab-scale glass reactor with an outside diameter of 2.4 cm and a height of 15 cm. It was used as the absorber, and 40 ml of prepared solution was fed to it each time before the absorption. JNYQ-I-41 infrared gas measurement analyzer from Xi'an JuNeng Instrument Co., Ltd., was used to measure NO concentration and was calibrated by passing the standard gases through the system each time before conducting the absorption experiments. The absorber inlet NO/N_2_ flow rate was set to 500 ml min^−1^, and the exhaust NO concentration was continuously monitored by the gas analyzer. The exit gas from the bubbling reactor was passed through an ice-water bath to avoid the effect of moisture on the analytical system.

All the absorption experiments were carried out at room temperature and ambient pressure. The NO concentration in the outlet was continuously monitored by gas analyzer with one-minute segments, and NO removal efficiency (*η*) was defined as follows:2
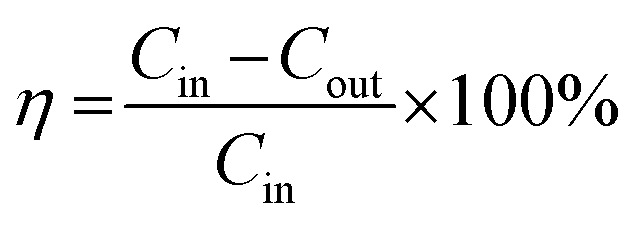
Here, *C*_in_ and *C*_out_ denote inlet and outlet NO concentrations (vppm) in the gas phase, respectively. As the absorbing solution regenerated, its absorption capacity would be recovered, which could be indirectly evaluated by the variation of *η*.

## 3. Results and discussion

### 3.1. Characterization of synthesized NZVI particles

**Fig. 2:**
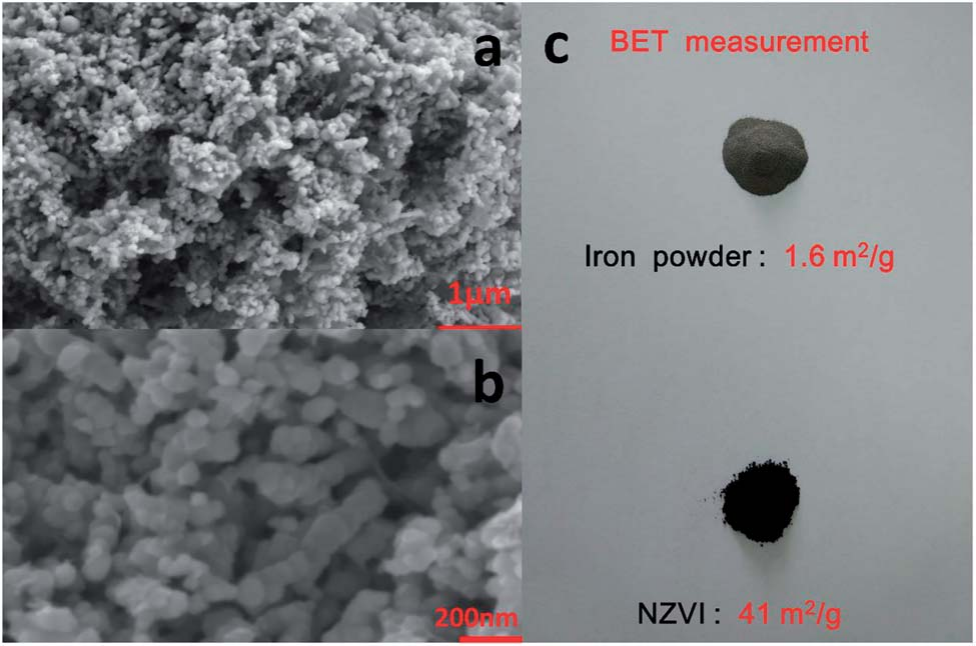
SEM images of NZVI with (a) 1 μm and (b) 200 nm scale bar; (c) photo image of reduced iron powder and the NZVI.

**Fig. 3:**
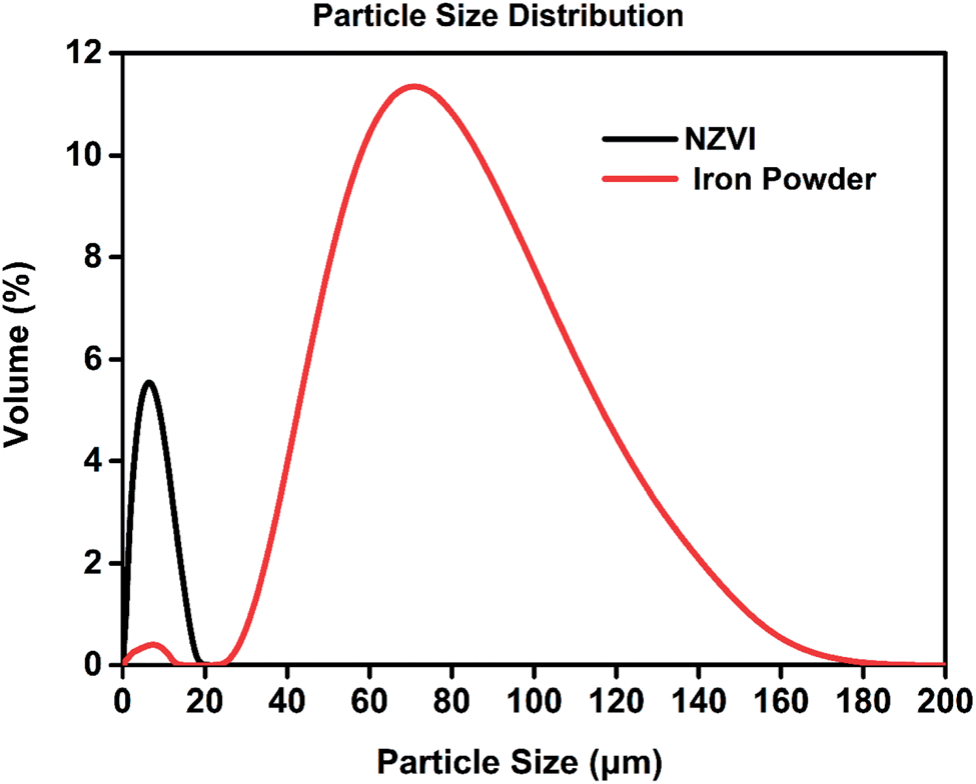
Particle size distribution of iron powder and NZVI in aqueous solution.

The morphology of the nanoparticles was investigated by field-emission scanning electron microscope (FESEM, Zeiss Merlin Compact) at 5.00 kV. As seen in Fig. 2, the synthesized particles are less than 100 nanometers in diameter and have a spherical shape, indicating successful formation of nanoscale particles, which has been described in Chen's work.^22^ As shown in the SEM images at different magnifications, the aggregation and stacking of NZVI were apparent and inevitable because of Ostwald ripening, arrested precipitation and direct inter-particle interactions.^24^ Afterwards, the surface areas of the synthesized NZVI and reduced iron powder were measured with nitrogen adsorption method at 22 °C. The results showed that NZVI had a 41 m^2^ g^−1^ specific surface area, which was about 25 folds larger than that of commercially available iron powder (1.6 m^2^ g^−1^). In order to quantitatively describe the size of these two particles in absorption experiments, the hydrodynamic diameters were measured using a Zetasizer Nano ZS (Malvern Instruments). According to the particle size distribution obtained (see Fig. 3), the size distribution of NZVI is narrow, below 20 μm, while the size of the reduced iron powder is spread over a wide area of 20–180 μm. Their median diameters in aqueous solution have been measured to be 4 μm and 64 μm, respectively. In all subsequent absorption experiments, these two particles with different sizes were adopted as additives to the absorption liquids.

### 3.2. Regeneration of Fe(ii)EDTA–NO by NZVI

The experiments involved the addition of the above chemical additive into NO-saturated Fe(ii)-EDTA solutions and instant regeneration before another NO absorption experiment was conducted. The revival of NO absorption would indicate that the reducing agent employed is effective for the regeneration of ferrous nitrosyl solution, by reacting with the bound NO.

**Fig. 4:**
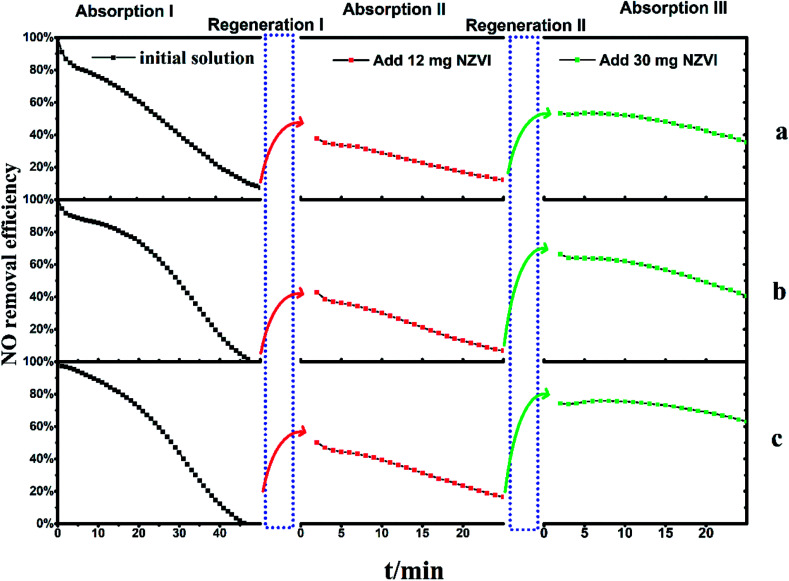
The two regenerations of Fe(ii)EDTA–NO solution with 12 mg and 30 mg NZVI addition, respectively. (a) pH 5.0 with acetate buffer, (b) pH 5.0, (c) pH 6.8.

The Fe(ii)EDTA solution initially showed favorable absorption ability under all the three different pH conditions. However, NO removal efficiency decreased gradually along with the introduced NO, which could be caused by the ferrous bonding sites being occupied by NO molecules to form the EDTA-Fe(ii)–NO species. As shown in Fig. 4, the exhaust NO concentration took approximately 50 minutes to reach a concentration consistent with the inlet, *i.e.*, a steady state of 1000 ppm. This indicated that the absorption process has been completed under all conditions. After that, the solutions saturated by NO, with EDTA-Fe(ii)–NO as the main composition, were regenerated by adding 12 mg NZVI in the bubbling reactor, and the re-absorption experiments (absorption II) immediately followed to examine the solution recovery levels. It could be noticed that NO removal efficiencies reached 38% (a), 43% (b) and 50% (c) at the beginning, and gradually dropped to 13%, 7% and 17% within 25 minutes, respectively.

In order to investigate the effect of regeneration times and the amount of NZVI added on NO absorption capacity, the above solutions, which have been absorbed twice and regenerated once, were fed with 30 mg NZVI for the next round of regeneration–absorption experiment. The results shown in absorption III (Fig. 4) revealed the NO removal efficiencies had improved to 53% (a), 66% (b) and 74% (c) at first and gradually declined to 36%, 40% and 63% within 25 minutes, respectively. These results suggest EDTA-Fe(ii)–NO could be regenerated more than once, and *η* could be improved along with the increase of the amount of NZVI added.

Another interesting phenomenon is that for these three solutions, *η* became more resistant to decline with the regeneration times, though the initial NO absorption capacity cannot be fully restored to the original value. Taking the solution with acidity of 6.8 as an example, the *η* decreased from 50% to 16% at a nearly constant rate after a gradual decline in the initial stage of absorption I. This period took 11 minutes, and the slope of the decrease was about 3.1% per minute. The absorption liquid was then regenerated by adding 12 mg of NZVI (regeneration I), and the absorption test was again performed (absorption II). The *η* also reduced from 50% to 16% at a constant rate, and the decrease slope of this segment became 1.5% per minute. Further addition of 30 mg NZVI for regeneration II and absorption III revealed that not only was the *η* greatly improved, but also the curve slope was further slowed down to 0.5% per minute, compared to that in absorption I and II. Therefore, it can be inferred that further increasing the addition amount of NZVI can significantly increase the denitration efficiency, and at the same time keep it stable for a long time, thereby facilitating the removal of NO. It is still unclear which variable, *i.e.*, the number of regeneration times or the amount of NZVI added, plays a key role in this result, as independent cross-testing has not been fully performed yet.

One of the most important factors controlling the regeneration is supposed to be solution acidity. The effect of pH on EDTA-Fe(ii)–NO reduction by zinc powder and iron powder has been investigated.^15,17^ Feiqiang and coworkers deemed that a low pH can promote the reduction of EDTA-Fe(ii)–NO by iron powder. Nitrosyl in solution was reduced into ammonium, releasing the active Fe(ii)EDTA, which was consistent with the reaction mechanism of the continuous consumption of H^+^.^15^

3 5Fe + 2Fe^II^EDTA − NO^2−^ + 12H^+^ → 2Fe^II^EDTA^2−^ + 5Fe^2+^ + 2NH_4_^+^ + 2H_2_O

4 Fe^0^ + 2H^+^ → Fe^2+^ + H_2_↑

Suitable acidity is indispensable because it can continuously provide hydrogen ions, which are consumed in large amount during the regeneration process, according to eqn (3). Meanwhile, the oxides and hydroxides attached to NZVI surface need to be dissolved by acid.^24,26^ The stronger the acidity of the solution, the faster the NZVI outer layer dissolves, which facilitates the regeneration–absorption process of the absorbent. However, hydrogen ions also accelerate the rapid corrosion and dissolution of NZVI in aqueous solution (eqn (4)), which is disadvantageous for the regeneration of the absorbent. It should also be noted that the stronger acidity has a negative impact on the ability of the Fe(ii) EDTA solution to absorb NO.^1,27^ In summary, the effect of solution acidity on the regeneration–absorption cycle is complex and multifaceted. In our experiments, the three pH conditions did not show significant differences and were all suitable for further investigation.

### 3.3. Expired Fe-EDTA solution regenerated by NZVI

The Fe(ii)EDTA solution would deteriorate gradually since the Fe(ii)EDTA is oxygen-sensitive. In pre-experiments, the freshly made solution lost its ability to absorb NO completely when exposed to air for three days. To examine the solution regeneration performance, the iron/NZVI-added solutions were fed to the absorber, and NO absorption experiments were conducted as described in the previous section. Specifically, the acidity of expired Fe-EDTA solutions was adjusted to the desired pH, and the solutions were then regenerated by 20 mg NZVI, 44 mg NZVI, and 100 mg reduced iron powder, respectively.

**Fig. 5:**
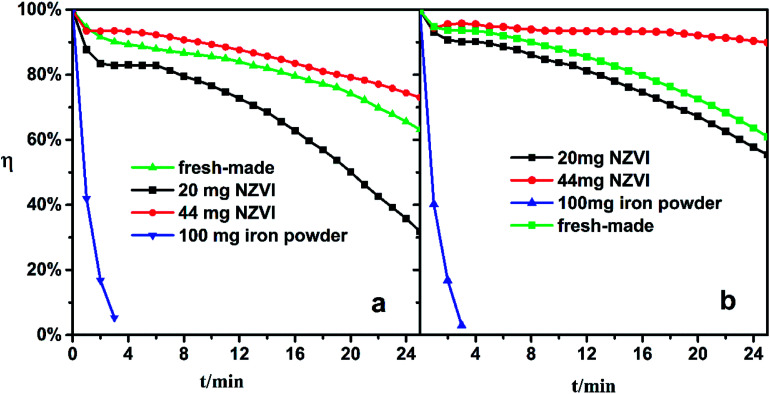
NO removal efficiency of expired Fe-EDTA solution with NZVI/iron powder addition. (a) pH 5.0, (b) pH 6.8.

Experiments show that the addition of iron powder has little effect on the denitration effect of the expired solution. As shown by the blue line in Fig. 5, the *η* quickly dropped to near zero in four minutes, which indicated rapid regeneration could not be achieved. Even if the iron powder is excessive, its specific surface area is too small to sufficiently contact with the inactive chelate component in the solution. However, the 20 mg and 44 mg NZVI-added solutions both showed satisfactory NO absorption performance under pH 5.0 and 6.8 conditions. The addition of 20 mg NZVI to the expired solution at pH 5.0 could keep *η* above 30% in 25 minutes, and 44 mg NZVI can restore the absorption ability as if the chelate solution were freshly made (green line). For an expired solution of pH 6.8, 20 mg of NZVI is sufficient to almost completely recover its absorption, and *η* not only recovers completely but also remains above 90% for a long time if the NZVI addition is increased to 44 mg.

It seems that the increase of the NZVI dosage not only benefits NO absorption performance, but also stabilizes *η* at a high level. This phenomenon could be attributed to two synergistic effects. On the one hand, more of the expired Fe-EDTA solution is regenerated with greater NZVI addition. On the other hand, more Fe(ii)EDTA chelates are released from the Fe(ii)EDTA–NO simultaneously, which are largely formed during absorption. Therefore, the conclusion obtained in the previous section is confirmed again.

### 3.4. Fe(iii)EDTA reduced by NZVI

It has been supposed that the oxidation of ferrous chelate to ferric chelate is the main reason for the deactivation of expired Fe-EDTA solution. To confirm this point, Fe(iii)EDTA solution was prepared to conduct similar NO absorption experiments to those described above. These experiments involved the use of a complex solution containing Fe(iii)EDTA and NZVI/iron powder to absorb NO. Since the ferric chelate does not coordinate with NO, positive NO uptake results will indicate that NZVI can effectively reduce ferric complex to ferrous complex.

It also needs to be mentioned here that [Fe^III^(EDTA)]^−^/[Fe^II^(EDTA)]^2−^ has lower redox potential (0.129 V) compared to [Fe^III^(H_2_O)_6_]^3+^/[Fe^II^(H_2_O)_6_]^2+^ (0.771 V),^28^ which narrows the reductants' selection range. Only strong reductants like dithionite ion and hydrazine, or active metals such as aluminum and zinc powder, could be considered as suitable candidates to reduce Fe(iii)EDTA effectively.^3,14,15^ According to references,^3,16^ the redox equation of Fe(iii)EDTA with iron powder or NZVI can be written stoichiometrically as:

5 2Fe^III^EDTA^−^ + Fe^0^ → 2Fe^II^EDTA^2−^ + Fe^2+^

**Fig. 6:**
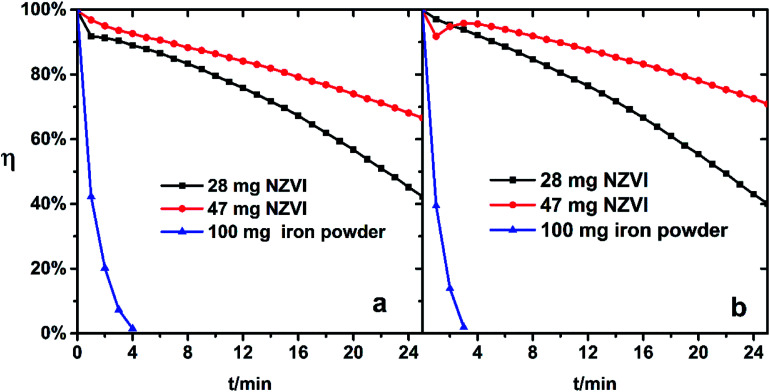
NO removal efficiency of Fe(iii)-EDTA solution with NZVI/iron powder addition. (a) pH 5.0, (b) pH 6.8.

If the above reaction to form ferrous ions and ferrous chelates can occur rapidly, the color of the absorbing liquid containing a large amount of ferrous species will change from brown yellow to dark green. This phenomenon was immediately observed after the addition of NZVI, but was not confirmed after adding iron powder for a while. The addition of 100 mg iron powder to Fe(iii)EDTA solution did not exhibit a better regeneration–absorption effect than that of 28 mg-added NZVI, as shown in Fig. 6.

This indicates that the specific surface and the size of the iron powder severely limit its reaction with ferric complex in aqueous solution.

Suitable solution acidity could be favored for the iron chelate to remain stable, prevent precipitation of iron compounds, and promote the complexation reaction of Fe(ii)EDTA with NO to some extent.^27^ But the increase of pH is unfavorable for the reduction of Fe(iii)EDTA.^16^ Therefore, the change of the solution from weakly acidic (pH 5.0) to neutral (pH 6.8) seems to have little effect on regeneration–absorption combination process, since the dual effects of acidity on regeneration and absorption offset each other.

**Fig. 7:**
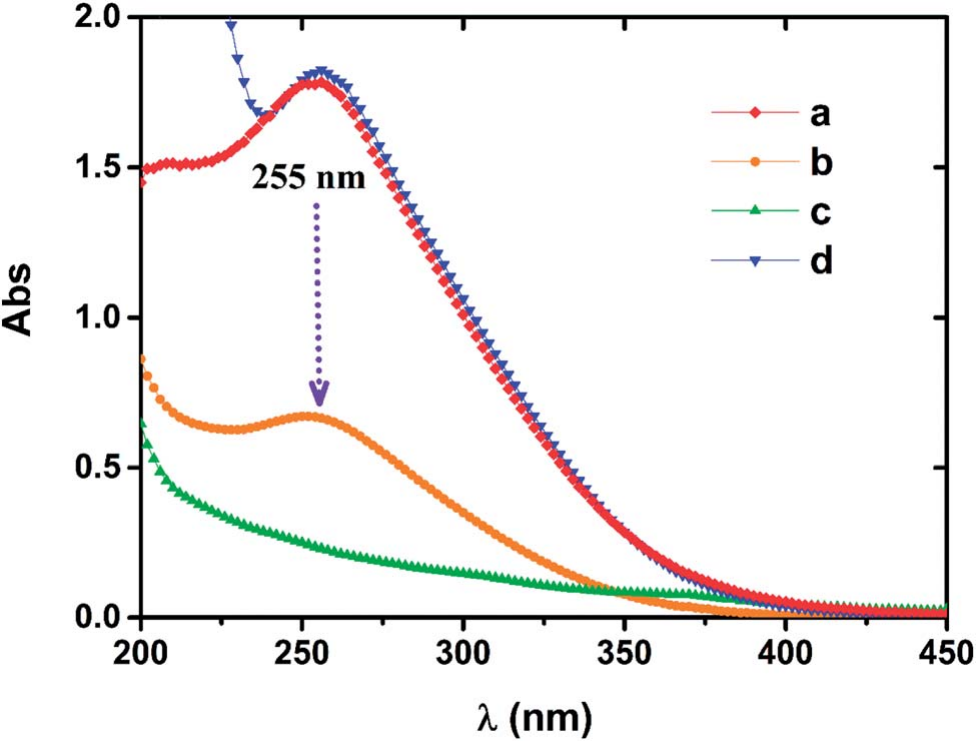
Characterization of UV-visible spectra of chelating solutions by UNICO UV-4802. (a) 0.2 mM fresh-made Fe(ii)EDTA solution in air; after adding NZVI for (b) 5 min and (c) 15 min, respectively; (d) UV-vis spectrum of 0.2 mM Fe(iii)EDTA solution.

The characteristic wavelength of 255 nm exists in both the Fe(iii)EDTA solution and the oxidized Fe(ii)EDTA solution, but this characteristic peak is not observed in the Fe(ii)EDTA solution under the condition of insulating air.^29^ When NZVI was applied to oxidized chelate solution, it was noted that the absorbance of the solution at 255 nm gradually decreased until it disappeared, and no new characteristic peaks were observed (see Fig. 7). This means that the ferric chelate is gradually reduced to the corresponding ferrous component. It further suggests that the central iron ions of the chelate only undergo redox reactions with the iron particles in the solution, and iron particles do not have a significant impact on the chelate structure itself. However, in long-term absorption–regeneration operations, the chelating agent EDTA is still likely to be slowly degraded by NZVI, as the latter can degrade other organic substances.^30–32^

## 4. Conclusions

A novel NO regeneration method has been developed based on NZVI technology. Compared to iron powder, the large surface area of NZVI gives it the ability to rapidly regenerate Fe(ii)EDTA–NO, expired Fe-EDTA, and Fe(iii)EDTA solutions. Solution acidity plays an important role in the regeneration–absorption process. NZVI reactivity and NO absorption rate all can be influenced under different pH conditions, and different solution acidity levels, from weakly acidic to near neutral, are all suitable for regeneration–absorption processes. Currently, with the development of new manufacturing processes, the cost of NZVI has been decreased greatly, which implies that the price of NZVI will be able to meet the requirements for both water and flue gas treatment in the near future.

## Conflicts of interest

The authors declare no competing financial interests.

## Supplementary Material
